# The Security of Big Data in Fog-Enabled IoT Applications Including Blockchain: A Survey

**DOI:** 10.3390/s19081788

**Published:** 2019-04-14

**Authors:** Noshina Tariq, Muhammad Asim, Feras Al-Obeidat, Muhammad Zubair Farooqi, Thar Baker, Mohammad Hammoudeh, Ibrahim Ghafir

**Affiliations:** 1 Department of Computer Science, National University of Computer and Emerging Sciences, Islamabad 44000, Pakistan; i131502@nu.edu.pk (N.T.); muhammad.asim@nu.edu.pk (M.A.); zubair.farooqi@nu.edu.pk (M.Z.F.); 2 College of Technological Innovation, Zayed University, Abu Dhabi 144534, UAE; Feras.Al-Obeidat@zu.ac.ae; 3 Department of Computer Science, Liverpool John Moores University, Liverpool L3 3AF, UK; 4 School of Computing, Mathematics and Digital Technology, Manchester Metropolitan University, Manchester M1 5GD, UK; M.Hammoudeh@mmu.ac.uk; 5 Faculty of Informatics, Masaryk University, 60177 Brno, Czech Republic; ghafir@mail.muni.cz

**Keywords:** security, big data, Internet of Things, fog computing, edge computing, blockchain

## Abstract

The proliferation of inter-connected devices in critical industries, such as healthcare and power grid, is changing the perception of what constitutes critical infrastructure. The rising interconnectedness of new critical industries is driven by the growing demand for seamless access to information as the world becomes more mobile and connected and as the Internet of Things (IoT) grows. Critical industries are essential to the foundation of today’s society, and interruption of service in any of these sectors can reverberate through other sectors and even around the globe. In today’s hyper-connected world, the critical infrastructure is more vulnerable than ever to cyber threats, whether state sponsored, criminal groups or individuals. As the number of interconnected devices increases, the number of potential access points for hackers to disrupt critical infrastructure grows. This new attack surface emerges from fundamental changes in the critical infrastructure of organizations technology systems. This paper aims to improve understanding the challenges to secure future digital infrastructure while it is still evolving. After introducing the infrastructure generating big data, the functionality-based fog architecture is defined. In addition, a comprehensive review of security requirements in fog-enabled IoT systems is presented. Then, an in-depth analysis of the fog computing security challenges and big data privacy and trust concerns in relation to fog-enabled IoT are given. We also discuss blockchain as a key enabler to address many security related issues in IoT and consider closely the complementary interrelationships between blockchain and fog computing. In this context, this work formalizes the task of securing big data and its scope, provides a taxonomy to categories threats to fog-based IoT systems, presents a comprehensive comparison of state-of-the-art contributions in the field according to their security service and recommends promising research directions for future investigations.

## 1. Introduction

Industries are adopting the emerging Internet of Things (IoT) paradigm and cloud computing services for the monitoring and controlling of critical applications associated with industrial control systems, smart grids, and other critical infrastructures [1,2,3]. In addition, industries are always concerned about the efficiency and how to reduce operational cost. They look for solutions that improve the performance, i.e., system stability, flexibility, fault tolerance and cost effectiveness, which may increase the interactivity and complexity [4]. The concept of IoT combined with cloud computing enables the systems to cater such industrial needs. The idea of IoT emerged as a result of interconnecting several kinds of physical devices [5]. The IoT provides users with innovative business models, modern embedded devices and better connectivity. The network through which these physical devices are interconnected lets them exchange data and communicates with each other. These devices can be sensors, smart meters, smart vehicles, smartphones, PDAs, Radio-Frequency IDentification (RFID) tags and any other device with embedded software [6]. Additionally, the automation is extended to the daily life of humans and lets IoT to be implemented in several domains such as smart homes, smart grid, smart city, e-healthcare, industrial automation, and intelligent transportation.

IoT devices generate a massive amount of confidential and security-sensitive data. Cisco expects that 50 billion devices would capture the Internet by 2020, and it will reach to 500 billion by 2025 [7,8,9]. It has also been predicted that the amount of generated data would approach 500 zettabytes by 2019. However, global data center’s IP traffic would merely reach 10.4 zettabytes [10]. However, cloud computing provides on demand storage and processing services for such kind of big data; there is a tradeoff between storage and latency. Most user-generated data are huge and need high bandwidth for transmission. Thus, moving the big data from the edge of the Internet to data centers, which are generally located near the core network and in places where operating cost is the lowest, is a challenging task [11,12,13]. Additionally, users’ experience with delay-sensitive applications such as real-time games, emergency services and human–computer interfaces, might be ruined when unexpected delays occur. Therefore, the importance of cloud computing cannot be ignored even for future innovations, yet there has emerged fog computing that might overcome the traditional drawbacks inherited from cloud computing such as latency issues, unavailability of location awareness, mobility support and bandwidth obstacles. Fog computing puts a substantial amount of communication, control, storage and management at the edge of a network as opposed to establishing dedicated channels to a more centralized remote cloud infrastructure. This approach reduces service latency, improves the Quality of Service (QoS) and provides a superior experience to end-users. It is estimated that fog computing would be used for 45% of IoT-generated data [10], and can be installed within the close range of IoT sensors and devices for local processing and data storage [14].

The Federal Trade Commission (FTC) Report on IoT urged business to adopt best practices to address consumer privacy concerns and security risks [15]. This report warns that smart devices are involved in harvesting huge amount of personal information and are exposed to a variety of potential security threats, such as unauthorized access and misuse of personal information. Data originating from ubiquitous devices are usually stored in cloud infrastructures, which not only attract intruders but also stakeholders, such as service providers and cloud operators interested to use these data for their own benefits (e.g., advertising) [16,17]. Fog computing can help to address some security concerns related to IoT-generated data. For instance, fog computing facilitates the on-site data storage and analysis of time-sensitive heterogeneous data by reducing the amount of confidential data stored and transmitted to the cloud. However, fog computing has its own privacy and security challenges due to low latency, proximity to IoT devices, decentralized architecture and transient support [8,18].

This research was conducted to understand the challenges to secure future digital infrastructure while it is still evolving. The key contributions of our study are as follows:

1. An in-depth analysis of the fog computing security challenges, big data privacy, and trust concerns was performed in relation to fog-based IoT along with their existing solutions and respective limitations.
2. It enacts the securing of big data with a novel functionality-based fog architecture taxonomy to categorize threats and security challenges in fog-enabled IoT systems accompanied by fog-enabled IoT applications security requirements. This provides a comprehensive comparison of state-of-the-art contributions in the field according to their security service.
3. Study of complementary interrelationship between blockchain and fog computing exploring blockchain-based solutions to cater privacy and security problems in fog paradigm along with the review of security requirements analysis of the fog-enabled IoT application with the combined blockchain.

This paper is structured as follows: Section 2 defines IoT and explores several IoT applications. Section 3 consists of fog computing generic and functionality-based architectures. Section 4 presents Fog-enabled IoT applications security requirements followed by fog computing security challenges in Section 5. In Section 6, we highlight interrelationships between blockchain and fog computing along with the blockchain-based security solutions in fog-enabled IoT systems. Finally, the conclusion is drawn in Section 7.

## 2. Data-Intensive IoT Applications

**Figure 1:**
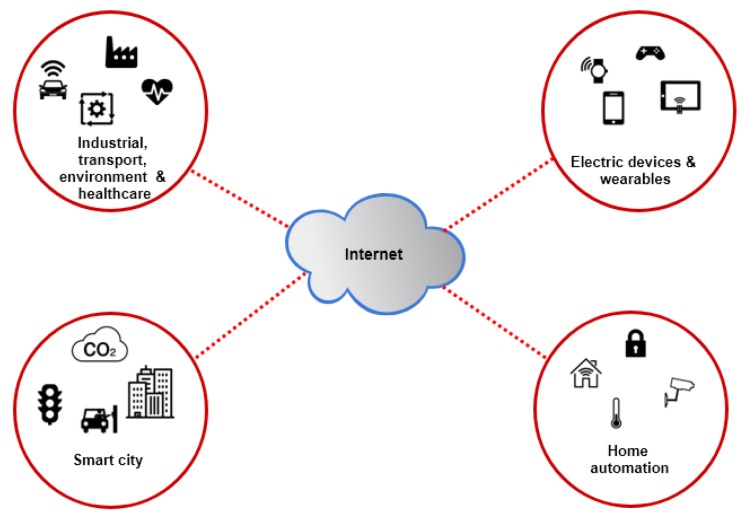
Data intensive IoT applications.

Any physical object that has the ability to connect over the Internet for data collection purposes with certain storage capacity and processing power is called a “thing” or IoT object. These “things” can be small sensors, mobile phones, gadgets, actuators or anything. Things are not only complex devices such as mobile phones, but can also be daily life things, such as food, a piece of art, furniture, landmarks, etc. IoTs have several areas of applications, from home automation to business functional areas such as logistics, transportation, health care, safety, intelligence and many morem as shown in Figure 1. Some of the most common data-intensive application are described below.

### 2.1. Smart Homes

Citizens are investing heavily in smart homes to save money and gain control of their homes and their lives. Smart homes use sensors and actuators attached to appliances and gadgets connected wirelessly to the home network and can communicate with each other to provide consumers with a seamless user experience. Various kinds of sensors installed in the homes constantly collect data from the surroundings and perform different intelligent home-related functions such as automatic turning on and off lights, appliances and gadgets, temperature control, security and many more. Sensors are heterogeneous, therefore the collected data are of various kinds. For example, sensors are installed to detect fire, humidity, gas leak, temperature changes, etc. If there is gas leak, for instance, it turns off the gas outlets automatically. There are also floor sensors installed to sense the pressure and track the movements of residents; in the case of any suspicious movement or falling of a person on the floor, they generate relevant actions. Smart homes also use surveillance cameras to record the activities or movements of the residents [8,19]. Energy conservation is another important function that can be achieved through smart home application [20].

### 2.2. Smart Cities

The smart city is a vast yet an important application of IoT [8,21]. It is implemented mainly to address specific issues related to citizens well-being such as traffic management, energy, transportation, and education. The smart city heavily relies on smart sensor devices to collect data such as temperature, humidity, pollution, and traffic conditions. For example, sensors are installed at the city water reservoirs and homes for the efficient utilization of water resources. Sensors installed at homes keep the record of the amount of water supplied, timings, pressure and other things. In addition, users can monitor the water meters for the billing purpose [8]. Smart traffic management is another smart city application where data collected from different sensors located in the city help in regulating the flow of traffic in response to demand. Such applications of the smart city lead to an exponential increase in data, which bring many problems and challenges, such as data security and privacy.

### 2.3. Smart Healthcare

Managing health services is another important task that has been automated using smart IoT-based e-health systems [22]. Technologists have developed many wearable smart devices, which monitor user’s overall health conditions. These devices keep the record of the patients’ health and generate alerts in the case of any abnormal activity. For example, smart e-Health services are proven very useful for the elderly and disabled individuals who find difficulty in movement. IoT systems can be deployed to remotely collect medical data (such as heart rate, physiological data, and blood pressure), and transmit these data to healthcare big data centers for storage and diagnosis [8,23]. Given the extreme sensitivity and confidentiality of the collected medical data, one of the most significant threats to healthcare services is that of data security and privacy [24].

### 2.4. Smart Environment and Agriculture

The technology has played a vital role in easing the daily life of the humans, yet there are dark sides which need to be addressed for their betterment. The smart environmental systems help in monitoring and controlling the environment. Air pollution has become a devastating issue around the globe. Smart environment applications use the sensors to detect the humidity, temperature, etc. Smart agriculture systems are used by farmers to get the greater yield of crops with better quality. The environmental parameters that are required for agriculture such as soil information, temperature, humidity, pesticide control, etc. are collected by sensors and sent to the servers. These parameters are analyzed in real time to guide the farmers about the condition of the soil and whether it is appropriate for the cultivation. Thus, it helps in producing the quality crops [8,25].

### 2.5. Energy Conservation

The smart grid is another useful yet complex application of IoT [26]. Smart grids have become the need of this time, which introduces a distributed and user-centric smart power grid system that aims to provide a reliable, efficient, secure and quality energy supply [27]. Smart grid technology comprises the two-way smart and intelligent flow of information is between consumer and the supplier. IoTs or sensors are installed at consumers’ place and the grid stations [28]. The sensors installed at consumers’ site are responsible to collect data related to electricity supply, consumption patterns, smart metering, pricing and other details. While the sensors at grid stations monitor the supply, distribution, discontinuity of supply, location identification, consumers’ information, etc. The data collected from those sensors are sent to their respective control centers where relative warnings are generated for smart and optimal distribution of electricity. A successful implementation of smart grid system requires explicit security and privacy solutions to maintain data confidentiality.

## 3. Fog Computing Architecture

**Figure 2:**
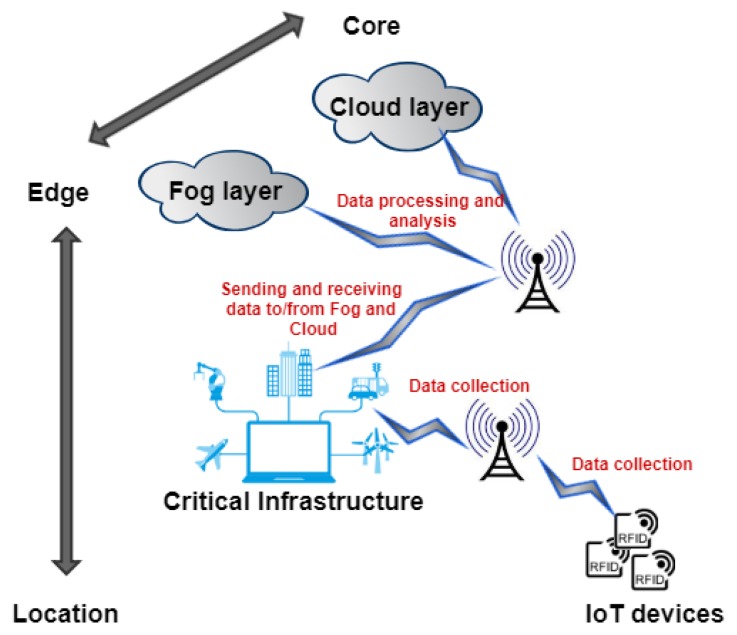
Fog-enabled IoT systems.

Fog computing provides decentralized on-demand services and applications for managing and analyzing big data directly at the network’s edge. It provides storage, processing, controlling and networking analogous to cloud computing. The fog layer serves as an intermediary between IoT devices and the cloud. The term fog-enabled IoT system is not new and has already been used in the literature [29,30,31]. For example, Byers [29] discussed some important architectural requirements for fog-enabled IoT systems along with a wide variety of potential IoT use cases, applicable to many vertical markets (i.e., transportation, utilities, smart cities, manufacturing, retail, agriculture, and health etc.). Azimi et al. [32] proposed a fog-enabled healthcare IoT system that is based on a three-layer architecture: sensor layer, fog layer and cloud layer. However, to the best of our knowledge, there is no standard architecture for fog computing [9]. We can broadly classify the fog-enabled IoT systems into three layers, as illustrated in Figure 2: IoT device layer, fog layer and cloud layer. For example, a fog-enabled IoT application of smart city collects data through various sensors, and offload some computations to the fog layer. This may also include the analysis and processing of delay-sensitive data at the fog layer. Moreover, if further computing and processing is required and it is not time crucial, the data can be sent to the cloud.

**Figure 3:**
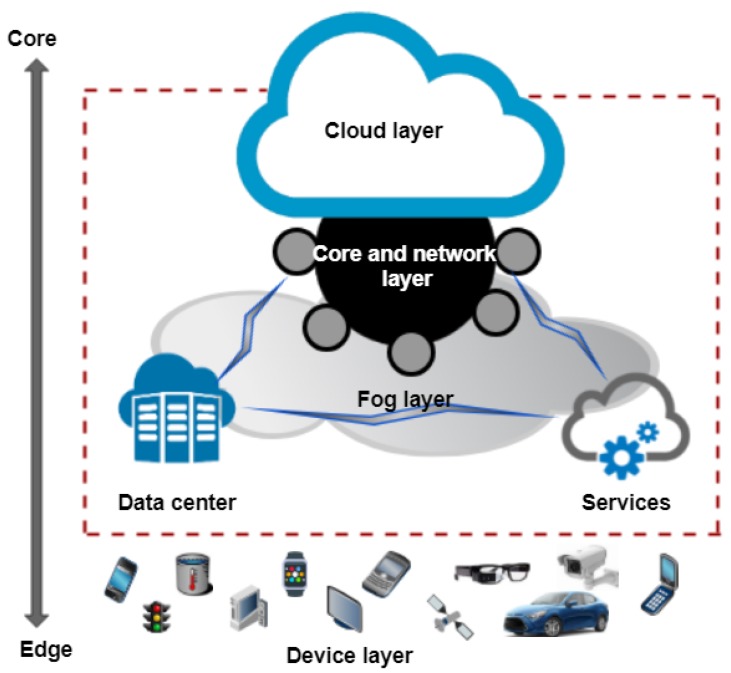
Functionality-based fog architecture.

In this work, we classified fog architecture into three distinct layers, which is inline with the architecture proposed in [16], as shown in Figure 3:

- Core-network and service layer
- Data center layer
- Device layer with inter and cross layer communication technologies

The core layer is responsible for providing network, management and other services to the end users. It encompasses fog nodes, e.g., routers, bridges, gateways, switch, base stations, etc. They are assisted with computing resources and local servers entailing controllers, embedded computing, smart phones and cameras. Network connections are used to deploy these fog nodes wherever needed, for example on roadsides, factory floors, power stations and in smart phones and vehicles. Infrastructure providers deploy data centers, which are found on edge of the network. Data center layer is responsible for multi-tenant virtualization infrastructure, which is used for scalability, flexibility and for enhanced computation, storage and other resource sharing requirements to meet user demands. It is used to achieve data and IoT application isolation and security for a concurrent and independent processing. Although these data centers are interconnected with the cloud layer through the network infrastructure, they can work and cooperate autonomously. The computing and storage capacity increases as we move forward from device to cloud in chronological order. There are two types of devices involved at the device layer: Mobile-IoT and fixed-IoT devices [8]. Mobile devices encompass peripatetic devices such as smart watches, smartphones, trackers, etc., whereas fixed-IoT devices have specific functions and remain stationary at particular locations, such as sensors and RFID tags. They are constrained in terms of storage and computing resources with limited bandwidth [33]. Due to their limited characteristics, they are not suitable for responding to emerging events and occurrences and are mainly used for data collection and submission to a higher layer.

Due to the underlying differences between cloud computing and fog computing, security solutions proposed for cloud computing may not suit fog services, which are available to end users at the edge of networks. This might impact the adaptation of fog-enabled IoT systems. Therefore, addressing security concerns at fog layer could enable fog paradigm to provide not only additional computational resources, but also adequate level of security to minimize cyber-attacks in fog-enabled IoT systems. Apart from fog security, the security of fog-enabled IoT systems is equally important for ensuring veracious data analysis and decision making. The security requirements and challenges in both fog-enabled IoT applications and fog layer are further elucidated in subsequent sections.

## 4. Fog-Enabled IoT Applications Security Requirements

As discussed in the previous section, fog-enabled IoT applications are going to be used in every field of life. These networks of smart devices are expected to be remote in nature and use wireless connection for communicating with other IoT devices or fog nodes [34]. This wireless communication medium is vulnerable to various network attacks, such as eavesdropping, etc. (Table 1 presents a list of different security threats to IoT-enabled applications). Thus, the most crucial security properties regarding data security are confidentiality, integrity, and availability. Confidentiality ensures adversaries do not gain unauthenticated and unauthorized access to data. Integrity refers to the completeness and accuracy of data. While, the availability of data and resources guarantees provisioning of network services and data to authorized users when required. In fog-enabled IoT applications, if these properties become compromised by attackers, the result may be devastating and disastrous. Another important factor in the security of IoT applications is the lack of standardized security. Mostly, IoT devices are manufactured by different vendors and the security of these devices lacks industry-accepted standards. Since many IoT security frameworks exist, there is no single agreed-upon framework. Big companies and industry organizations may have their own precise standards, while certain sectors have proprietary, incompatible standards from industry leaders. The variety of these standards makes it difficult to not only secure systems, but also to ensure interoperability between them. Furthermore, authorized entities may start misbehaving to exploit IoT devices with respect to offensive data manipulation, false data injections and so on. Besides that, IoT applications are largely deployed as Low power and Lossy Networks (LLN), such as wireless sensor networks, smart city, and smart health applications. The LLN are a class of networks where the interconnected devices are highly resource constrained (power, memory, processing, etc.), and are characterized by high loss rates, low data rates and instability in the communication links [35]. Conventional cryptography and trust-based security protocols consume power, storage and computing resources and may result in message overhead and low trust convergence. In IoT environment, existing solutions do not seem to be effective in the sense of security and protection against internal attacks. Table 2 presents a list of internal attacks on the fog-based IoT applications routing mechanism. Moreover, Existing security mechanisms do not take into account the impact of device mobility, which is essential in scenarios such as smart city, smart health, etc. The interconnecting devices such as gateways or field devices can also easily be targeted by hackers from various network communication interfaces. Therefore, all smart devices, for example in a smart home environment, need to be secured with strict security mechanisms; otherwise, an attack on any individual device may result in the malfunctioning of the whole network [36].

IoT systems can be established by connecting sensors with a distributed data transmission system for remote access, processing and storage. Thus, the biggest threat in this working environment is the data security breach, which is often high due to its complex nature at rest and in transit. For example, Liu et al. [37] discussed the significance of secure data transmission among WLAN-based IoT applications. Moreover, the architecture of IoT devices make it difficult to embed security solutions in every device. Therefore, the need of integrating the fundamental security controls in devices and applications arise while considering the ecosystem where these devices are used. The major challenge is the privacy and security of the data generated by home sensors and detectors. The collected data are stored at multiple locations, e.g., on the connected device itself, in the cloud, at the edge, or in on-premises infrastructure. Researchers and cyber security experts are routinely uncovering vulnerabilities in smart homes IoT devices, which could lead to unauthorized access to consumer data, and compromise consumer privacy, security and safety [8,38]. In smart city domain, secure interactions and verification are also prodigious security concerns [39]. It should support the digital forensic investigation among the connected components [36] along with implementing and maintaining the end-to-end security and privacy supports for data acquisition, transmission and processing [40]. The system should be equipped with lightweight cryptography-based policies for working in an environment where the resource-constrained IoT devices are in abundance [41].

The health care related IoT applications generally deal with patients personal data, which are subject to data breach if proper security measures are not in place. The devices used in these applications are generally low in processing and storage, due to which they cannot integrate additional security protocols. In addition, these applications are mobile in nature (i.e., they may require to be connected to any public network such as home and office), thus making these applications more vulnerable to tempering and forgery attacks. The increasing number of IoT devices have also made it difficult to design a dynamic and stable security system that can guard against all the possible security threats [42].

Similarly, smart grids are the hot points for security and privacy attacks during collecting, receiving and sharing data. If an attack is successful, it may result in the deterioration of government services such as telecommunication companies, energy distribution and other associated services [43]. The loss may be in the form of data or service impairment. The main source of these attacks is the unauthorized access to the network, which results in the alteration or destruction of database.

## 5. Fog Computing Security Challenges

Cloud computing has significant weaknesses to protect its computing framework and storage from malicious attacks [62,63]. Some of the security and privacy threats are summarized in Table 1. Fog-based architectures are more secure than cloud architectures for several reasons including less dependency on the Internet and possible storage and information exchange between the cloud and its users in non-real-time [64]. Fog-enabled IoT systems employ various networks for interconnecting all the participating devices, such as wireless and mobile core networks making them potential targets for any attack [65]. Network monitoring can be helpful in detecting anomalies and security vulnerabilities [66].

Therefore, the analysis of these top layers becomes more critical from security point of view. Furthermore, in some of fog-enabled systems, the core infrastructure is implemented by same party who manages these centers [16,67,68]. A virtualization infrastructure in edge data centers can be integrated to give the ability for deployment of cloud at the network edge. The biggest threat in this system is potential attacks on virtual machines [69,70,71].

In the real-world, the data centers are a hub of virtualization servers along with other managerial services controls. From security prospective, the whole edge data center is at great risk as it includes all public APIs, which are responsible for providing the services to connected users and other access points such as webs applications [72,73]. Individual IoT devices are also the rudimental targets for security attacks as they actively participate in data transmission [74,75]. However, the extent of damage by a compromise of these devices is very limited as they can influence only the network segment they are attached to, which makes them low-risk compared to the previously discussed threats.

**Figure 4:**
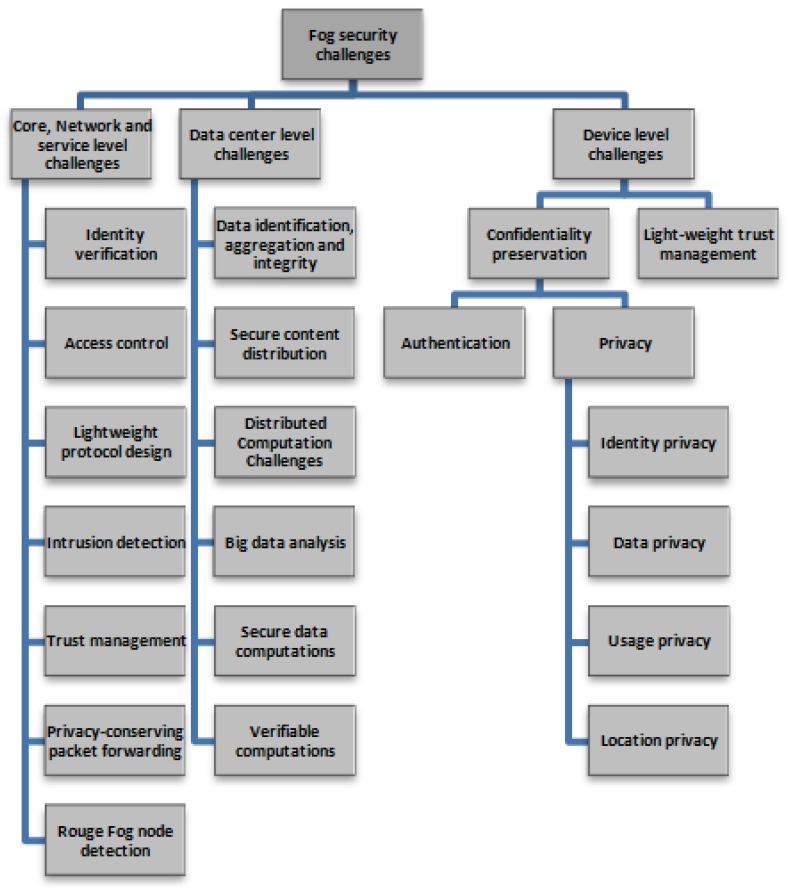
Taxonomy of IoT-based fog security challenges.

Fog security challenges are divided into three major classifications, as described in Section 3: (a) core-network and service level; (b) data center level security; and (c) device level. Figure 4 shows a taxonomy of these security challenges and the following subsections present detailed discussions of these challenges.

### 5.1. Core-Network and Service Level Challenges

The core-network and service layer leads to maintain a hierarchical multi-tiered architecture. The underlying core infrastructure, such as mobile core infrastructure or centralized services makes it easy to manage and register various services [16]. As in cloud, fog also has capabilities to provide storage and processing of data collected from local IoT devices. With this facility, the fog can handle real-time local computational services. Specifically, IoT applications and services are offered by the fog nodes, which are installed at the network edge to control activities as well as decision-making tasks based on the data collected from IoT devices with the response time of milliseconds [76]. Therefore, it is possible to develop several delay-sensitive IoT applications that can be used for quick decision-making activities while collecting local data from these IoT devices. Smart traffic lights, healthcare and activity tracking, and decentralized vehicular navigation are a few of the delay-sensitive IoT applications that are fog assisted.

There are several proposals in the literature to secure core-network and overcome real-time service challenges, which are summarized in Table 3 and discussed below.

#### 5.1.1. Identity Verification

Identity verification is referred to as the authentication of a legitimate IoT device within an IoT network to use fog-based services. IoT devices must verify their identities to avail fog services in a secure manner. Several authentication schemes are proposed in the literature [9,77,78]. These schemes support IoT services and build secure fog computing environment. An effective technique is the cooperative authentication mechanism [8], which focuses on the minimization of authentication overhead by removing redundant authentication messages to reduce the delay caused by the authentication process. In real-time services, another challenging issue is related to user privacy. To fulfill user privacy requirement, anonymous authentication mechanisms have been adopted to authenticate users without revealing their identity [79].

#### 5.1.2. Access Control

Access control determines permissibility of certain network resources to only those devices/users who possess certain rights to use the requested resource. Authorization is just as important as authentication in fog computing. Due to heterogeneity and poor security controls in IoT devices, it is often easier for attackers to gain access to these devices. Salonikias et al. [80] proposed a role-based authorization in traditional web services in which administrators have to regulate access rights. In [81], attribute-based encryption is proposed, in which predefined attributes of users must be satisfied [82,83]. However, there are open challenges related to multiple devices for predefined policies.

#### 5.1.3. Lightweight Protocol Design

In real-time services, short-range communication between IoT and fog nodes one or two hops away may have a significant impact on the overall system performance. Due to the resource-constraint nature of IoT devices, there are several lightweight protocols designed to resolve these runtime issues. The authors of [84] provided multiple lightweight cryptography schemes including hash functions and stream chippers for secure end-to-end communication.

#### 5.1.4. Intrusion Detection Challenges

Many intrusion detection techniques have been proposed in the literature to reduce the success of attacks such as flooding, insider attacks, pot scanning, and attacks on a virtual machine and on hypervisor in the cloud system. Intrusion detection mechanisms monitor a network for distinguishing between malicious and benign nodes/activities. These mechanisms monitor and analyze user login information, log files and access control policy to identify any malicious activity on the network. For secure fog, intrusion detection is one of the important elements. There are several proposed works such as the host-based intrusion detection system [27], which collects from the cloud information including systems calls and file systems and then analyzes it. Arshad et al. [85] proposed a model for intrusion detection with minimal human intervention and response time. Hamad et al. [86] introduced intrusion detection system as a cloud service. Houmansadr et al. [87] proposed a mobile phone-based intrusion detection system.

#### 5.1.5. Trust Management

Although identity verification and access control create a trustful connection between fog nodes and IoT devices, to procure authenticated misbehaving nodes projecting insider attacks still pose a challenge. Some of the important internal attacks are discussed in Table 2. A certain trust levels among fog network devices is needed as well as a resilient security mechanism to prevent such internal attacks. Hence, trust-based mechanisms are widely used to cater such issues. There are so many definitions given to trust in the literature. Here, trust is defined as an acceptance level between two nodes for a defined action. The trust value is used to imitate whether a sensor node is eager and able to perform normally. A threshold is set for the node to be tagged as good or bad. The range of the trust value is mostly between 0 and 1 [88]. Often, 0 refers to completely malicious and 1 is the opposite. For mobile social networks, an investigation of trustworthy user’s evaluation service was proposed by [89] to mitigate the capabilities of Sybil attacks. There might be multiple trust levels of fog nodes. Therefore, for real time services, there is no cooperation between patterns behaviors, which causes a lack of trust management. Different trust management schemes are adopted in [90,91] to analyze different trust management models. Wei et al. [92] proposed a direct evidence-based trust management scheme. Su et al. [93] contributed an attribute-based trust management scheme.

#### 5.1.6. Privacy-Conserving Packet Forwarding

Data packets that are received either from IoT devices or other fog nodes are forwarded through fog nodes, hence it is mandatory to preserve privacy of forwarded packets [8]. Resource constrained and mobile nature of IoT networks cause distinct packet forwarding features in fog-enabled IoT system. End-to-end connectivity is difficult, thus cooperation of other nodes is required for message delivery. Selfish nodes may become reluctant to be a part of message forwarding due to consumption of limited resources such as energy. Moreover, malicious nodes may collude to be the part of the route to perform malicious activities on data packets [94]. Therefore, privacy-conserving packet forwarding is a must. There are many security solutions such as remote data integrity verification, which allows the user or other trusted party to verify the data integrity. Data encryption before uploading on fog node by IoT devices is a fundamental requirement that secures the data from privacy leakage. Encryption of data by IoT devices causes an obstacle to share data with trusted parties. An atomic proxy cryptography scheme was proposed by Blaze et al. [77], in which semi-trusted proxy convert cipher-text by a sender is decryptable by a receiver without watching the original plain text through proxy encryption key. Blaze et al. [77] proposed chosen cipher-text-based secure proxy re-encryption. Canetti et al. [95] proposed a conditional proxy re-encryption that meets various data sharing requirements. For secure data sharing, the authors of [96] proposed attribute-based re-encryption in which secret key assignment is based upon attributes of the user. The authors of [97] proposed Ciphertext-Policy Attribute-Based Encryption (CP-ABE) and Key-Policy Attribute-Based Encryption (KP-ABE) schemes with a difference of associated access policies. Intermediate networks are utilized for IoT devices and cloud communication to build two-way communication by fog nodes. End-to-end security is a basic requirement to prevent sensitive data to disclose during transmission. It degrades properties of data sharing such as aggregation, search, and sharing.

#### 5.1.7. Rogue Fog Node Detection

In fog environments, IoT devices transmit data to fog nodes, which process those data to provide services to the users. The workload is divided and assigned to several fog nodes to be processed in the case it is increased. A fog node is considered as rogue fog node when it acts as a legitimate node, but it is rather a compromised node. Hence, ensuring data integrity becomes a difficult task if any fog node is compromised by malicious activity. Therefore, the trust among fog nodes must be ensured before any data processing and computation starts. This requires an authentication protocol that may enable trust among nodes. In the case there are many data to be processed, fog nodes that are authenticated by the cloud should not be located anywhere outside the fog environment. The authors of [98,99] demonstrated the possibilities of man-in-the-middle attack where the gateway is swapped by fake or compromised one. The existence of a compromised node in the environment can be a threat to data integrity, security and privacy of user information. There are several reasons that such issues are difficult to address, among which the most important ones are: (i) trust management schemes to cater complex trust situations; and (ii) difficulty to maintain the list of rouge nodes due to creating and deleting Virtual Machine (VM) instances dynamically [98]. There are different attack resilient trust-based routing mechanisms to help in detecting rogue fog nodes [100,101].

### 5.2. Data Center Level Security Challenges

The fog is equipped with resources that can be used for storing big data collected from IoT devices temporarily. This makes the data readily available to be frequently accessed by users, as well as helpful in maintaining and updating the data in a well-organized and flexible way. This temporary storage on fog nodes significantly reduces the delay in communication between the fog and the cloud and, hence, cuts down the response time to access and update the data. Fog data centers act as the intermediate devices in networks taking up the responsibility of performing different communication functions such as data collection and assembling, packet transfers and routing. The fog data centers are also capable of simple processing of the received data and can select the appropriate audience for data distribution. Fog data centers serve the purpose of implementing a multi-tenant virtualization setup. These systems work under the control of infrastructure providers. These data centers are used by every user including consumers and infrastructure providers. These edge data centers work autonomously while cooperating with other connected devices, but they are also interconnected with traditional cloud centers. Thus, there is a possibility to implement multi-tiered architecture using network infrastructures for network formation. For the implementation of this system, we have to evaluate the existing support infrastructure systems such as mobile core networks and centralized cloud services. The fog-based systems are heterogeneous in nature, which allows high speed wireless connections and technologies to work together [16,102]. Large-scale data collection and distribution is expressively optimized because of the involvement of fog nodes. Fog nodes and IoT maintain temporary data as a transient storage to reduce big data management complexity. It is also very crucial to safeguard data distribution and dissemination. However, some security and privacy issues seriously affect data privacy. There are several challenges and proposed solutions found in the literature; some are surveyed in the following subsections and are summarized in Table 4.

#### 5.2.1. Data Identification, Aggregation and Integrity

IoT devices generate huge amount of data, but not all of the data are useful. These devices are unable to identify and distinguish sensitive and important data out of the generated data. Sensitive data identification, aggregation and integrity are critical challenges for fog-enabled IoT systems before uploading to data centers. Data must be aggregated as big data are being generated and collected by several IoT devices and identified as sensitive and required data before processing. Although it is difficult for IoT devices to identify sensitive and useful information, it is possible to classify IoT devices having sensitive data. Furthermore, there is an important requirement to temporarily maintain the data in transient storage to minimize management complexity. However, there are some privacy issues, which affect data confidentiality, integrity and sharing. Ghafir and Prenosil [103] presented a novel mechanism to identify malicious downloaded data. To achieve aggregation, there are several techniques presented in the literature. In [104,105], the authors proposed Paillier encryption, which is widely implemented for smart grids. Lu et al. [106] designed an efficient data aggregation scheme for fog-assisted IoT devices through homomorphic Paillier encryption. In [78,81], a homomorphic scheme is adopted to achieve encrypted data processing. For searchable encryption, the authors of [107,108] adopted different privacy levels to make searchable data without information exposure. Yang et al. [109] proposed a location-based services to limit user access for out of range areas. Boneh et al. [110] achieved encrypted mail that is searchable on unreliable mail services. Iovino et al. [111] implemented an encrypted data search based on multiple keywords.

#### 5.2.2. Secure Content Distribution

Information leakage due to content distribution services is controlled by multiple states of the arts. The authors of [112] proposed a secure discovery scheme to ensure authorized user identification for the discovery of information. Park et al. [62] suggested a broadcast encryption scheme by delivering encrypted information at the broadcast channel. To protect data privacy at fog nodes, decentralized computing is adopted to secure data from attacks. Papamanthou et al. [113] proposed a verifiable computational scheme based on content-based encryption mechanism. Choi et al. [79] proposed a model design for dynamic computation at fog environment.

#### 5.2.3. Distributed Computation Challenges

Fog computing is capable of transient storage and computing, where several fog nodes can perform decentralized data computation cooperatively. Fog nodes can take up cloud computational tasks and act as proxies to help users in performing heavy processing tasks. Computation offloading, aided computation, and big data analytics are some of the important fog assisted IoT applications where decentralized computation is involved [114]. Fog nodes can perform certain computational activities such as processing of data and analysis. Due to potential security breaches in fog computing, the data to be processed may be available to attackers to gain control of the computational results and it may compromise fog node performance. Hence, ensuring data privacy becomes a huge concern for users when they assign computations to fog nodes.

#### 5.2.4. Secure Big Data Analysis

Big data is the amalgamation of many technological revolutions that aim at addressing the processing capabilities and storage capacity of systems. Furthermore, the large volume of heterogeneous data are being analyzed using big data analytics that employs many advanced and parallel data analysis techniques. Fog nodes collect big data for processing and analysis in the form of text, images and videos. However, the data analysis techniques also pose many threats regarding the privacy and security of personal data as the data are obtained, stored and analyzed on any available online sources. Thus, the Internet users are prone to privacy infringements, as their personal data can be used in any form, for any type of analysis, and these tools are capable of extracting sensitive and important information. To counter security issues, several traditional techniques for data security are being employed. However, they are unable to counter these problems due to the complexity and heterogeneity of big data [115]. Therefore, there is a need for developing new and advanced security systems. To address such challenging issues, e.g., securing users data privacy and analyzing data instantaneously, fully homomorphic encryption [116] and differential privacy are commonly applied. Li et al. [116] proposed cloud-aided privacy-preserving frequent item set mining scheme for vertically divided databases. Xu et al. [117] advocated the use of certificate-less proxy re-encryption scheme that uses randomized and re-encryption keys for data sharing while restricting the control over privacy settings and trust on cloud environment. A Hilbert curve-based cryptographic transformation technique is presented in [118] to protect privacy along with improved querying process for outsourced databases. The data confidentiality along with query result integrity is guaranteed in the approach of Jang et al. [119], who employed a privacy-aware query authentication process.

#### 5.2.5. Secure Computation

Users do not have full control over computations in distributed environments. This generates privacy and security concerns. For this reason, it is necessary to guarantee secure computations. In this regard, Matsumoto et al. [120] introduced the concept of server-aided computation. Their ultimate target was to use insecure auxiliary devices for speeding up private computations. Numerous server-aided computation protocols (e.g., [121]) are introduced. Girault and Lefranc [122] proposed the concept of server-aided verification for speeding up the process of authentication/signature verification by deploying a portion of computation to untrusted yet powerful servers. Later, a generic procedure achieved server-aided verification using bilinear maps.

#### 5.2.6. Verifiable Computation

Verifiable computing offloads the computations to some other perhaps untrusted servers on the condition that results are verifiable and valid. In fog computing, the cloud offloads computations to the fog in distributed fashion. In addition, due to lack of computation resources, the users also approach fog nodes to submit computations for local processing. The fog is not completely trustworthy and may return incorrect results [8]. Thus, for the both cloud and the user, it is mandatory to check correctness of computed results or it may result in computation offloading failure. Gennaro et al. [123] designed a scheme of non-interactive computation and proposed the concept of verifiable computation. Using a small size of a public key, Chung et al. [124] applied homomorphic encryption schemes and constructed non-interactive verifiable computation scheme. Based on CP-ABE, Parno et al. [125] designed a scheme for publicly verifiable computation. To verify dynamic computation in the cloud environment, Papamanthou et al. [113] introduced a new model. Choi et al. [79] applied proxy oblivious transfer schemes to support multiple users laid down multi-user non-interactive verifiable computation scheme.

### 5.3. Device Level Security Challenges

In fog computing environments, each device has unique identity and visibility. Not all IoT devices are resourceful; many of them have constrained resources such as limited power, storage and computational capacities. Thus, they send their data to upper layer (fog/cloud as required) through gateways for processing, storage and provisioning of different services. Fog-based IoT systems bring significant privacy and security concerns [126]. The following crucial properties must be considered for securing the data produced at the device level, which are presented and outlined in Table 5.

#### 5.3.1. Confidentiality

Confidentiality ensures that data are not exposed to an unauthorized entities. There is a great computation and storage overhead involved in existing security protocols such as SSL/TSL-based communication [127]. For example, it is not effective to apply existing PKI-based systems on all IoT devices due to their heavyweight computation and storage. The PKI and CIA protocols provide strong security in terms of fixed and predefined large sized keys, therefore causing memory and processing overhead. They also do not cater for insider attacks [128]. Cloud architecture does not meet IoT devices requirements such as timing, scalability and location awareness [129]. Therefore, lightweight infrastructure is crucial to provide a timely response.

1. **Authentication:** As fog computing services are offered to an enormous number of end users through front end fog nodes, authentication becomes a critical issue. Fog nodes require authentication at different levels to ensure security in fog computing, as explained by Stojmenovic et al. [102]. The existing conventional PKI-based authentication is unable to overcome the security issue being less efficient and with lower scalability options. On the other hand, there exist simple, user-friendly and secure solutions to cater the authentication issues in a location limited channel while depending on physical contact in local ad-hoc wireless network [130]. Moreover, biometric authentication has emerged as an important technology when it comes to authentication in mobile computing, cloud computing and fog computing. Fingerprint authentication, touch-based authentication or face authentication are few examples [98].
2. **Privacy:** Users are getting more concerned about the breaching to their private and sensitive information such as personal data, location or other information while using the cloud services, IoT or wireless networks. Therefore, it is the most crucial challenge to preserve the privacy in distributed fog environment, as the fog nodes operate at user’s end and must gather more sensitive data than the cloud. Several researchers have proposed various techniques to preserve the privacy in different setups such as wireless network, online social network [131], smart grid [132] and cloud [98,132].
  - **Identity privacy:** IoT user’s identity must be protected and preserved from public and other IoT user to prevent impersonation attacks. Different pseudonym techniques [133,134,135] have been proposed to preserve identity privacy. However, periodic pseudonyms may lead to heavy computation cost in resource constraint IoT domain. Furthermore, group signature [134] and connection anonymization [136,137,138] techniques are also proposed for protecting identity privacy [94].
  - **Data privacy:** The algorithms to preserve privacy in fog networks run between cloud and fog, but the fact that these algorithms utilize a huge amount of resources at the edge devices cannot be ignored. Fog nodes collect sensitive data that are generated through end devices and sensors [98]. At local gateways, homomorphic encryption can be employed without decryption to permit privacy-preserving collection [139]. Another technique that differential privacy [140] is employed in the case of statistical queries to ensure the privacy of uninformed data entries.
  - **Usage privacy:** Fog computing comes with another very important concern of users’ usage pattern privacy. For instance, the smart meter in smart grids reads and collects a huge amount of data that are private to users, such as at what times user is unavailable at home, the consumption pattern, switching on and off certain appliances, etc.; such information is a threat to users’ privacy. Many researchers addressed privacy preserving techniques in smart metering [141,142,143]. It is unfortunate that these techniques cannot be employed in fog computing because of unavailability of a trusted third-party device to cater energy limitations. One approach to preserve privacy is to create fake tasks by fog client and send them to other nodes; in this way, the real tasks are hidden behind fake ones. However, this solution is inefficient in terms of cost and energy consumption. Therefore, an effective approach is to design a solution that divides the application in a way that ensures the usage of distributed resources minimizes the disclosing private information [98].
  - **Location privacy:** The term location privacy denotes the privacy of the location of fog clients in fog computing. When a fog client divests the task to fog nodes, it assumes that those nodes are located nearby, and other nodes are distant, though this is not always the case. Moreover, the fog client may use several fog services at different locations, there are fair chances that the path trajectory may be disclosed to fog nodes. The location privacy is at risk as long as the fog client is attached to a person or device [98]. One way to hide the location of the fog clients is to obfuscate the fog client identity, so that even the fog node knows that client is nearby and still unable to locate it. Wei et al. [144] proposed several techniques to obfuscate the identity, one of which is to employ a trustworthy third party which may generate false identities for each fog client. In real scenarios, it is not necessary for a fog client to choose the nearest fog node, but even if it does so, the client may undergo some criteria such as reputation, latency or load balance to reach that node which may utilize more resources than usual. This can lead the node to have an idea of clients’ location but not in a precise manner. Gao et al. [145] proposed a method to preserve the privacy of client’s location in such situations.

#### 5.3.2. Light-Weight Trust Management

The crux of fog-based IoT network is to endow secure and trustworthy services. There is a need to have certain trust levels among fog network devices. Authentication plays a crucial role in setting a trustful connection between fog nodes and IoT devices. It becomes indispensable to answer what if authenticated nodes behave maliciously such as provoking insider attacks. Conventional trust-based routing protocols have message overhead, high power consumption, require more memory and low trust convergence. Therefore, there is a need to design a trust-based mechanism that is lightweight with low processing, delay and memory consumption overhead and effective in identifying misbehaving nodes. Khan et al. [146] proposed a trust-based resilient routing mechanism for IoT which was not effective enough with bad/good mouthing attacks and supported only routing protocol for low power and lossy devices. A comprehensive trust-aware routing protocol with multi-attributes for WSNs was proposed by Sun and Li [101], which is characterized by complex calculation processes. For energy efficient trust-aware routing protocol for WSNs, [147] proposed several solutions influenced energy paths problems created by dishonest watcher, slow trust convergence and longer paths.

## 6. Blockchain: A Versatile Security Solution

The concept of blockchain was coined in 2008 [148] for bitcoin cryptocurrency network. Therefore, bitcoin is strongly supported by blockchain that is an unassailable log, which keeps record of network transactions. The blockchain incorporates a distributed ledger to accommodate each and every transaction of underlying network. Participants on network, manage blockchain in a distributed method by using variable Public Key (PK). The new records are added in blocks through a process called mining via certain nodes that are known as miners. The process of bitcoin mining comprises of Proof of Work (PoW), which is actually a cryptographic puzzle that consumes the enormous amount of resources.

Because of the key features of blockchain such as anonymity, decentralization and security, it is very useful technology to cater to the above-mentioned security and privacy problems in fog-enabled IoT systems in an easy, efficient, trustworthy and secured manner. In addition, it has been widely implemented for provision of authorized identity to IoT devices. The decentralization ability of blockchain ensures security, authentication and integrity of transmitted data by IoT devices to be cryptographically proofed, assigned by authentic sender using unique public key and GUID (Global Unique IDentifier). Blockchain has made easy the secure tracking of any IoT device transaction [149]. There are many domains such as (but not limited to) eHealth [150], smart home automation [151], authentication and secure communication [152], and data preserving and integrity [153] where blockchain has a remarkable impact, as shown in Table 6.

Another very useful feature of blockchain is smart contracts which provide effective rules to authenticate the IoT devices, with nominal complexity as compared to conventional protocols for authorization. Furthermore, smart contracts are also capable of providing privacy to data either in transit or at rest with already set rules and conditions to allow the access to single or multiple users. Furthermore, smart contracts are useful in sensing and averting malicious actions. The system refuses the breached blockchain updates of the device. It also eliminates centralized entity where devices do not need any centralized device for their secure communication. Instead, they can securely communicate with each other, share information and automatically perform executions with the help of smart contracts. Another advantage to implement blockchain is that it provides unique GUID and symmetric key pair to each IoT device connected to blockchain network which completely omits the process of key management and distribution. This simplifies the other security protocols as well, as there is no need left for exchanging PKI certificates or creating master and session keys for the purpose of encryption coding parameters at handshake phase. This way, feasibility of light-weight protocols increases as it would fit the memory resources requirement of IoT devices. In addition, it provides secure communication among devices that enable the verification of device’s identity validity and ensure verified cryptography of the transactions made by authentic user. Moreover, there is no single failure point as identical information is recorded on several computers and devices [126,154].

Apart from above-mentioned advantages, there are a few challenges associated with using blockchain in IoT domain [155,156]. In many instances of IoT devices, adaptive and lightweight blockchain security solutions are necessary due to constrained computational and storage capacities. The computations regarding PoW may be disregarded in this scenario. In addition, bitcoin blockchain also poses latency in terms of response time for transaction validation and hence is not suitable in real time domains. Moreover, as discussed above, IoT devices generate huge amount of data as the result of numerous transactions, which consumes colossal amount of bandwidth consequently. Even though blockchain allows anonymous transactions, Conoscenti et al. [156] discussed that pseudonyms still may be pursued. Furthermore, in consideration of IoT growth-rate, bitcoin blockchain will eventually face scalabilty issues. The blockchain is also prone to security hazards; the most common attacks are 51% and majority attacks [157,158].

### Blockchain and Fog-Enabled IoT Systems

Fog-based IoT systems bring significant privacy and security concerns. The big data produced by a huge number of interconnected IoT device are sensitive and confidential. Thus, it is inevitable to provide end-to-end security and trust. The introduction of blockchain in fog-enabled IoT systems can solve such problems. The design of distributed fog services should be accompanied with state-of-the-art security systems that are capable of working autonomously in real time. As fog computing possess a distributed computing environment, it is impeccable to use distributed security mechanisms to secure network resources and data transactions, such as distributed trust and security solutions. Therefore, blockchain technology offers good grounds for fog-enabled IoT systems to build and manage distributed and decentralized trust and security solutions. The independent consensus among fog nodes and blockchain security architecture secure the network when the new device connects itself to the fog network. Furthermore, it can also detect and isolate the malfunctioning or compromised node to protect the whole system from any security breach. Therefore, this provides the much needed self-healing capability to the fog-enabled IoT systems. For complete success of IoT systems, it needs a new and efficient mechanism of security as the traditional security system and mechanism are unable to cope the challenges posed to this architecture. Therefore, industrial cyber-security systems should come with data securing mechanism to protect big data along with active corporation and control among connected devices [164,165].

Moreover, all the fog-enabled IoT applications store large quantity of data for different purposes, such as enterprises store data related to their customers for observing their trends related to their specific interest which causes the privacy concerns. Among this, most of the issues arise due to intervention of third-party apps and services. Blockchain systems can provide highest possible security measures in this regard. The most important feature of blockchain is the introduction of secure storage and transmission by digitally signed documents for enhanced protection and privacy. Moreover, this technology may possibly offer an effortless infrastructure to directly transfer data among IoT devices for secure communication through a reliable time-stamped contractual handshake. These applications are widely used in trade and finance departments due to their sensitive nature for identity validation of users [166]. There exists some state-of-the-art systems in IoT domain [151,167,168,169]. For the smart city, Sharma and Park [170] proposed a hybrid network architecture combined with Software Defined Networking (SDN) and blockchain. The authors also proposed a blockchain based scheme for distributed vehicular networks in [171]. In [159], they used SDN and blockchain technology for IoT based transport management system using a distributed mesh network infrastructure. For big data, BigchainDB [158] was used for increased throughput and to cater latency issues with the help of decentralized blockchain system. A literature review on applications of blockchain is presented in [156], mentioning the usability of blockchain in IoT domains. The authors also highlighted integrity and adaptability issues of blockchain in IoT systems. Samaniego and Deters [172] compared both cloud and fog platforms to justify which performs better for blockchain based IoT networks. According to their findings, the fog-based scenarios outperform the cloud-based IoT systems.

The security system equipped with blockchain-based security satisfies all the requirements of fog-enabled IoT systems by enhancing independent operation between all the connected nodes. It provides all the required qualities such as distribution and heterogeneity, which is the same as thos provided by the blockchain-based systems. However, fog applications are not supported by all blockchain consensus mechanisms, for instance Proof of Work (PoW) cannot be hosted on fog devices as it demands enormous resources such as power and computing to execute transactions. The OpenFog Consortium [164] aims at expeditious fog computing adoption and interoperability with the blockchain technology.

## 7. Conclusions

Due to lack in hardware/software security designs and constrained resources, IoT devices are vulnerable to different security attacks. This paper discusses potential security and privacy challenges in fog-enabled IoT system. The main goal of this work is to provide insight on securing big data generated by fog-enabled IoT applications. We started with different IoT applications that generate massive amount of data followed by fog computing architecture, fog-enabled IoT applications security requirements and fog computing security challenges. We studied different existing state-of-the-art security and privacy approaches to map these challenges along with their limitations. In addition, we also considered the blockchain as an emerging security solution along with the potential benefits to address security issues in fog-enabled IoT domain accompanied by some existing blockchain solutions in IoT systems.

Pursuant to our study and findings, we suggest that existing cryptographic and PKI mechanisms are not appropriate and suitable for resource constraint IoT devices such as sensor tags. There should be efficient security mechanisms that do not exhaust such devices in terms of computation, storage and energy. The blockchain is a decentralized security mechanism, which ensures security, authentication and integrity of transmitted data by IoT devices to be cryptographically proofed. It also provides features such as anonymity; it is a useful technology to cater to security and privacy problems in IoT in an easy and efficient way. Although blockchain provides many advantages, there should be efficient lightweight blockchain security proposals that do not exhaust resource constrained IoT devices in terms of computation, storage and energy. 

References

## Figures and Tables

**Table 1:** Security and privacy threats in IoT-enabled applications.

Threat	Description
Forgery [44]	Fake identities and profiles, fake information to mislead the user. Saturate resource consumption through fake data. That is, in E-Health and home automation systems, one can easily fake their identifications and profiles to generate any attack.
Tampering [45]	Degrading the efficiency of fog by dropping/delaying transmitting data. That is, energy conservation systems are responsible to collect the data related to electricity supply, consumption patterns, smart metering, pricing and other details. As the data are very critical, dropping or delaying the data may cause problems.
Spamming [46]	Spreading redundant information which causes to consume resources unnecessarily. The attack generated on smart cities lies in this domain.
Sybil [47]	Legitimate user personal information and manipulation of fake identities to take over the illegal control on fog resources. That is, in smart home and smart cities, legitimate user can manipulate the fake identities to take control of the network.
Jamming [48]	Jam communication network by spreading burst if dummy data on the network. Any type of smart environment can be attacked by Jamming.
Eavesdropping [49]	Capturing of transmitting packets and try to read the contents. Any type of smart environment can be a victim of these attacks.
DoS [50]	Flooding of superfluous requests to fog nodes to disrupt the services for users. The data generated by smart cities and smart agriculture can be a victim of DoS and flooding attacks.
Collusion	Acquiring unfair advantage through deceiving, misleading and defrauding legal entities by collusion of two or more parties.
Man-In-The-Middle [50]	Involving between two parties and manipulate exchanged data between them. E-Health and Smart cities are the best fit examples.
Impersonation [51]	Pretending the fake services as fog services to the users.
Identity Privacy [52]	User personal information leakage such as phone number, visa number, etc. on a communication channel.
Data Privacy [52]	Exposure of user data to unreliable parties considerably reaches to privacy leakage. Smart homes, smart cities and E-Health systems are commonly known victim of these types of attacks.
Usage Privacy [52]	Leakage of services utilization pattern of users.
Location Privacy [52]	Capturing user’s location information to expose or observe user moments. Smart homes, smart cities and E-Health systems are commonly known victims of these types of attacks.

**Table 2:** Internal attacks on fog-based IoT routing.

Attacks	Description
Wormhole [53]	Initial attacked node forms a path by colluding with other nodes to transfer malicious packets. The path formed among conspiring nodes is called wormhole.
Blackhole [54]	A malicious node intervenes in route discovery to be a part of path. It then drops the packets instead of forwarding them. Some blackholes attack the received packets before forwarding.
Greyhole [54]	A modified version of blackhole attacks. Data are dropped by the attacking node, but it tells the router that data are transmitted. This attack is difficult to detect by the router as it shows end-to-end connectivity.
Selective forwarding [55]	Selective data packets that are required to be transmitted are dropped by nodes resulting in network performance degradation.
Local repair [46]	Destabilizing the network and draining neighbor nodes battery by sending false link repair messages. It reduces packet delivery increases end-to-end delays.
Route cache poisoning [56]	It involves the alteration of route tables by malicious nodes to poison route caches to other nodes.
Sybil [47]	Assumptions of nodes to have multiple identities over the network to create confusion and disruption, which opens the opportunity for malicious nodes to operate.
Sinkhole [57]	Malicious node pretends to be the optimal route to the destination node by sending false messages to the initiator node, thus after receiving traffic, it alters the routing and other data to complicate the topological structure of the network.
Hello flood [58]	The attacker node broadcasts links to other nodes. The unsuspecting nodes accept that link and consider the attacker node to be the neighbor node. The unsuspecting node start sending packets that are actually wasted as the adversary node is far away and not the neighbor. This creates a routing loop within the network.
Neighbor [54]	The neighbor node considers the attacking node (while broadcasting DIO messages with no DIO details) as a newly joined node, which could be a parent node, but this node is out of reach when the neighbor node tries to select it as a parent node.
Version number [54]	The attacker node alters its version number in DIO messages and broadcasts to neighbor nodes. This results in routing loops in the network, which disrupt the network topology and deplete nodes energy resources.
Modification [59]	Malicious nodes take advantage of no trust levels being measured in the ad-hoc networks to engage in discovering, altering and disrupting the routing in the network. This attack causes traffic redirection and DoS attacks by modifying the protocol messages.
Fabrication [60]	Creates forged routing information using routing table overflow attacks, resource consumption and fake route error messages.
Byzantine [61]	Aims to decline the network services; the attacker node selectively drops route packets, which create routing loops and send forward those route packets through non-optimal paths.
Location spoofing [61]	Pretends to be the nearest destined node to disrupt normal network protocol operations.

**Table 3:** Core-network and service level challenges and solutions with respective limitations.

Challenges	Solution	Limitations
Identity Verification	Identity authentication Co-operative authentication Anonymous authentication	Difficult identity authentication realization due to decentralized nature of fog computing Difficult to manage the increased number of users Authentication overhead and delays due to the mobility of IoT devices Redundant authentication efforts are needed Cooperation of fog nodes is needed Privacy preservation is needed Response delay is not acceptable for real time services
Access Control	Role-based Access Control policy Attribute-based Access Control policy Device and key management	Strong credential handling policies are needed to ensure trustworthiness Federated and distributed access control architecture is needed due to mobility and dynamic device management by the user Multiple device management is needed when accessed by a single user There must be consistent access policy for each user employing different devices to access the services Key management is needed
Lightweight protocol design	Lightweight cryptographic techniques Lightweight elliptic curve cryptosystem	Need to design efficient lightweight protocols to support real time services of fog assisted IoT applications
Intrusion Detection	Host-based Intrusion Detection system (IDS) Network-based IDS Distributed IDS Mobile Sybil defence Cryptography-based Sybil defence	It is challenging to design a robust, reliable and efficient IDS for fog computing due to its heterogeneous, decentralized and distributed architecture Local as well as global intrusion detection systems are needed in fog computing Behavior features sharing is needed among cooperative fog nodes; the way information is shared in a decentralized architecture to obtain quick detection of intrusion and its prevention is a challenging matter The basic information of the user is needed by the detector to differentiate between legitimate and Sybil user resulting in privacy leakage In Sybil Defence, the data available on single fog node might not be enough to know if the user is Sybil or not because of crucial cooperation among fog nodes
Trust Management	Evidence-based trust model Monitoring-based trust model Reputation management	Behavior information of fog nodes is difficult to collect and maintain in order to maintain the trust evaluation of fog computing in decentralized architecture Situational trust matrices are needed for various services and applications Adaptive, scalable and consistent trust management design is needed due to IoT device mobility
Privacy-conserving packet forwarding	Privacy-preserving packet forwarding	Users privacy leakage
Rogue fog node detection	Trust-Based Routing Mechanism	Complex calculations Scalability issues Message overhead Slow convergence

**Table 4:** Data center level challenges and solutions with respective limitations.

Challenges	Solution	Limitations
Data identification, aggregation and integrity	Symmetric encryption Asymmetric encryption Homomorphic encryption One-way trapdoor permutation Key distribution and key agreement Homomorphic signature Provable data possession	Overhead of identifying sensitive data Difficult to protect sensitive data due to the large number of IoT devices Data aggregation requirements vary due to heterogeneous IoT application. Difficult to check the integrity of data due to transient storage, user mobility and variety of keys used by IoT devices Data integrity verification is comparatively less efficient
Secure data distribution	Proxy re-encryption Attribute-based encryption Key-aggregate encryption	Due to time-consuming bilinear pairing, secure data sharing is not very efficient Key management is challenging
Secure content distribution	Secure service discovery Broadcast encryption Key management mechanism Anonymous broadcast encryption	Key management and broadcast encryption is challenging Simultaneous secure service discovery and anonymous broadcast encryption is needed
Secure big data analysis	Fully homomorphic encryption Differential privacy	Computational overhead Designing decentralized big data analysis is challenging with differential privacy
Secure computation	Server-aided exponentiation Server-aided verification Server-aided encryption Server-aided function evolution Server-aided key exchange	Execution of complex computational tasks heavier than exponentiation, encryption/decryption and signature verification Smaller multiple fog nodes are even powerful than a single server
Verifiable computation	Privately verifiable computation Publically verifiable computation	Mostly based on theoretical approaches Due to distributed architecture of fog computing, an error may be spread to other nodes resulting incorrect final results Verification of results is needed Tracing of compromised fog node is needed

**Table 5:** Device level security challenges and solutions with respective limitations.

Challenges	Solution	Limitations
Confidentiality	Authentication protocols Privacy preservation techniques	Difficult identity authentication realization due to decentralized nature of fog computing Scalability issues Response delay due to the mobility of IoT devices Memory and processing overhead due to fixed and predefined large sized keys High computation cost Key management is needed
Light-weight trust management	Trust-based routing protocols	Trade-off between computation cost and security requirements Scenario specific Compatibility issues with resource-constrained IoT devices

**Table 6:** Blockchain-based security solutions in IoT systems.

Description	Advantages
A distributed IoT network architecture consisting of an SDN base network using the blockchains technique [159]	Improved system’s performance and capacity Threat prevention and protection, data protection, access control, and mitigate network attacks such as cache poising/ARP spoofing, DDoS/DoS attacks
An efficient decentralized authentication mechanism based on the public blockchain, Ethereum to create secured virtual zones for secure communication [152]	Not limited to specific IoT services and scenarios Relies on a public blockchain, hence possesses all of its security properties Well defined security requirements for authentication
A lightweight BC-based hierarchical architecture for IoT that uses a centralized private Immutable Ledger and a distributed trust to reduce the block validation processing time [149]	Lightweight yet retains privacy and security benefits of classical blockchain security solutions Elimination of overheads associated with conventional blockchain No mining and its processing related delays Low packet and processing overhead
A decentralized network model based on blockchain approach for data preserving, data integrity and blocking of unregistered devices using Physical Unclonable Functions (PUFs) and Ethereum [153]	Unique identity to each IoT device Defense against botnets and bogus requests such as Denial of Service (DoS), and Distributed Denial of Service (DDoS) Data provenance and integrity
A blockchain-based decentralized, infrastructure-independent proof-of-location technique for location trustworthiness and user privacy preservation [160]	Unlimited identifiers for users to avoid location attacks Blockchain is used to store proofs of location Geographic location verification User location privacy preservation
A cloud-based blockchain solution for identifying IoT devices manufacturing provenance while enforcing users privacy preservation using EPID (Enhanced Privacy Identity protocol) of Intel to incentivize IoT devices for data sharing [161]	Support anonymous device commissioning and incentive to IoT devices Ensures privacy-preservation
A blockchain-based scheme called Healthcare Data Gateway (HGD) architecture to enable patient to own, control and share their own data easily and securely without violating privacy [150]	No need for trusted third party Ensures privacy-preservation Ensure data confidentiality, data authenticity and data integrity
A blockchain-based security and privacy scheme for smart homes [151]	Low packet, time and energy overheads Ensured availability of devices Resilient against DDoS and linking attacks
A blockchain solution for preserving data privacy in Internet of Things using smart contracts along with a firmware scheme using blockchain for prevention of fraudulent data [162]	Trustless access control management constrained IoT device tampering to prevent fraudulent data
A blockchain-based proof of concept for securing consumer/home-based IoT devices and the networks by using Ethereum [163]	No significant storage and CPU overheads Utilization of built-in asymmetric key encryption and digital signatures present in Ethereum protocol
